# The Role of Cyclooxygenase-2 in Colorectal Cancer

**DOI:** 10.7150/ijms.44439

**Published:** 2020-04-27

**Authors:** Juan Sheng, Hong Sun, Fu-Bing Yu, Bo Li, Yuan Zhang, Ying-Ting Zhu

**Affiliations:** 1 Department of Gastroenterology, the Second People's Hospital of Yunnan Province, Kunming, Yunnan 650021, China; 2 The First Affiliated Hospital of Nanjing Medical University, Nanjing, Jiangsu 210029, China; 3 Department of General Surgery, The Second People's Hospital of Yunnan Province, Kunming, Yunnan 650021, China; 4 Tissue Tech Inc, Miami, Florida 33032, USA

**Keywords:** cancer, colon, cyclooxygenase-2, invasion, prostaglandin

## Abstract

Colorectal cancer is the third common cancer in this world, accounting for more than 1 million cases each year. However, detailed etiology and mechanism of colorectal cancer have not been fully understood. For example, cyclooxygenase-2 (COX-2) and its product prostaglandin E_2_ (PGE_2_) have been closely linked to its occurrence, progression and prognosis. However, the mechanisms on how COX-2 and PGE_2_-mediate the pathogenesis of colorectal cancer are obscure. In this review, we have summarized recent advances in studies of pathogenesis and control in colorectal cancer to assist further advances in the research for the cure of the cancer. In addition, the knowledge gained may also guide the audiences for reduction of the risk and control of this deadly disease.

## Introduction

Colorectal cancer (CRC) is a common fatal cancer in developed countries such as USA 1, 2, accounting for more than 1 million cases each year 3 and 700,000 deaths 4. Most recently, it is reported that colorectal cancer ranks fourth in the world's deadly cancer, accounting approximately 900,000 deaths yearly, due to increase of aging populations, obesity and lack of exercise 5. In fact, colorectal cancer (CRC) is the second most frequent deadly cancer in the United State. The proposed causative factors include but not limited to genetic mutations and chronic inflammation. Until recently, it is still not known what causes CRC because of complexity of the etiology of CRC.

Etiologically, colon cancer is caused by spread of colorectal cancer cells to other parts of the body. The causative factors include genetic alterations, overexpression of Cytooxgenase-2 (COX-2), smoking, drinking of alcohol, harmful diet and lack of physical exercises 6-8. Among those ricking factors, the occurrence of the cancer is closely linked to overexpression of COX-2, which has been noted in most of CRC 9, 10. Increased levels of COX-2 mRNA and protein are found in the great majority of colorectal adenocarcinomas compared with levels in adjacent histologically normal mucosa 9. A causal role for COX-2 in colorectal carcinogenesis is demonstrated *in vivo* in a murine model, and the biologic effects of upregulation of the enzyme are mediated predominantly through increased PGE_2_ production 11. Therefore, many researchers have been trying to use COX-2 inhibitors such as nonsteroidal anti-inflammatory drugs (NSAID) and COX-2 inhibitors (COXIB) to control this deadly disease.

In this review, we summarize recent advances in understanding of COX-2 signaling in etiology of CRC. We also try to renew our interest in prevention and control of colorectal cancer by NSAID and COXIB.

## COXs

COXs are important regulators of angiogenesis, inflammation and carcinogenesis. COXs are located at luminal side in the endoplasmic reticulum and associated with the nuclear envelope 12, containing three isoforms, that is, COX‐1, COX-2 and COX‐3 13. COX-1 is a housekeeping enzyme to meet the basic requirement for prostaglandins (PGs) 13, 14. COX-3 is a variant of COX-1 mainly within central nervous system 15, 16. In contrast, COX‐2 is an inducible isoform 17 in normal tissue such as colorectal, kidney, reproductive organs and stomach 18, 19. However in carcinogenesis, COX2 can be constantly upregulated 17, 20, for example, adenocarcinoma, squamous cell carcinoma, cholangiocarcinoma, endometrial carcinoma and hepatocellular carcinoma 21, 22.

Many factors, for example, DCA, IL-1β and LPS might promote expression of COX-2 moderately in normal fibroblasts (NFs), but profoundly in cancer-associated fibroblasts (CAFs) 23. Our results have clearly demonstrated that COX-2 is enhanced by DCA, HGF and IL-1β 24-27. As a result, Prostaglandin E_2_ (PGE_2_) production is greatly promoted, and such promotion increases proliferation and invasiveness of epithelial cancer cells 25, 27, 28. Nevertheless, COX-2 inhibitors such as NS398 may decrease proliferation and invasiveness of colorectal cancer cells by overexpression of COX-2 and its product PGE2 25, 27, 28.

## Stromal Cells in Colorectal Carcinogenesis

Stromal cells, for example, fibroblasts actively participate in carcinogenesis 29. We have reported that fibroblasts from the stromal compartment play a pivotal role in COX-2 signalling and carcinogenesis 25-27, 30. As shown previously, cancer-associated fibroblasts (CAFs) may promote epithelial ovarian cancer 31. Cytokines, for example, IL-1β, Tumor Necrosis Factor-α (TNF-α) and other compounds, for instance, deoxycholic acid (DCA) stimulates COX-2 expression, which enhances PGE2 production in colorectal fibroblasts 32-38. In addition, COX-2 expression and PGE_2_ production in CAFs from biopsies of colorectal cancer tissues are much greater than those from normal fibroblasts (NFs) 33. Therefore, we should focus on the mechanism how COX-2 expression and PGE2 production is medicated and how such findings are linked to progression and invasion of colorectal cancers.

## PGEs and Their Receptors

COX-2 is an enzyme regulating PGE_2_ within our body 39. Prolonged PGE_2_ increase is usually a sign of inflammation, cancer genesis and spread. COX-2 mediates biosynthesis and release of prostaglandins using arachidonic acid (AA) as the substrate 39. In other words, this enzyme first converts arachidonic acid into prostaglandin G_2_ and prostaglandin H_2_, and then synthesize prostaglandin D_2_, E_2_, F_2_α, I^2^ and thromboxane A_2_, exerts their actions through the cognate G-protein-coupled receptors (GPCRs) 39.

Prostaglandins are active lipid compounds which have multiple hormone functions to participate in inflammation and progression of colon cancer 40, 41.

Prostaglandin signaling is involved in the progression of many diseases including chronic diseases such as cancer, suggesting prostaglandins are indeed associated with regulation of both acute and chronic inflammation 9. The main form of prostaglandin involved in colorectal cancer is PGE_2_. PGE_2_ can act on the receptors, for example, EP1, EP2, EP3, and EP4 to induce PGE_2_ signal cascade, leading to changes of intracellular calcium, cAMP and some inflammatory factors. As a result, physiological or pathological processes follow 23, 42. Recent investigations support that PGE_2_ may enhance progression of colorectal cancer 41, 43, 44, and EP4 is a therapeutic target for cancer therapy 45, 46. COX-2 derived PGE_2_ can also contribute to tumor development through several mechanisms including inhibition of apoptosis. However, the mechanisms by which PGE_2_ regulates apoptosis are still largely unknown. The EP_2_ and EP_4_ receptors mediate their activities through cAMP production. Suppression of apoptosis has been seen in intestinal cells by cAMP through the induction of the IAP family member-inhibitor of apoptosis 2 (IAP-2) 47, 48 Therefore, it is wise to investigate the hypothesis that anti-apoptotic effects of PGE_2_ are mediated through cAMP, which results in the induction of the IAP family member c-IAP2.

## The cellular distribution of COX-2 and PGE_2_

The sub-epithelial tissue lies immediately underneath the epithelial layer. The mesenchyme of a tissue lies immediately underneath the epithelium. That is, fibroblasts are the predominant cells of the sub-epithelial layer. Interestingly, the cellular site of COX-2 upregulation in the earliest intestinal cancer tissue is sub-epithelial, and not epithelial.

In fact, recent studies have demonstrated that fibroblasts play a far more varied role than you anticipate. The cells express many receptors for cytokines and hormones and modulate intestinal secretary responses to inflammatory mediators by releasing PGE_2_.

The sub-epithelial site of inducible COX-2 expression in early murine intestinal adenomas conflicts with alternative evidence that, rather, upregulation of this enzyme occurs in colorectal epithelial cells 49. To reconcile the issue, we hypothesize that in normal and premalignant colorectal tissue, mesenchymal cells are the principal source of COX-2 expression. Nevertheless, once malignant transformation has occurred, the enzyme is also expressed in the epithelium. The question, then, arises of which mesenchymal cells might express COX-2 in nonmalignant colorectal tissue.

### COX-2 signaling is a marker for tumorigenesis

COX‐2 is a short-lived rate‐limiting enzyme 19, 50. It converts arachidonic acid (AA) to prostaglandins (PGs) and thromboxanes 51. A major PG product of COX-2 is PGE_2_, which may regulate angiogenesis, immunity and tumorigenesis 52-54. In carcinogenesis, COX-2 may be uncontrollably elevated, transcriptionally or post‐transcriptionally 55, 56. Therefore, increase of COX-2 expression is a marker for tumor diagnosis, which is associated with patients' survival rate 17, 57-60. The significant roles of COX2 signaling has closely been correlated various types of cancer as reported 13, 18, 61-63. Recent research suggests that PGE_2_ may indeed significantly promote development and progression of colorectal cancer 41, 43, 44. COX‐2 is also critical to promote metastasis 64 participating in metastasis to bone 65, brain 66, liver 67 and lymph nodes 68. COX-2-medicated Factors like IL‐11 is associated with cancer metastasis 65. Metastasis occurs when cancer cells invade extracellular matrix, blood vessels and finally lymphatics 69. Growing evidence has indicated that COX-2 signaling plays important roles in colorectal cancer metastasis. For instance, transactivation of EGFR by Src can promote the invasion mediated by PGE_2_ 70. PGE_2_ may also promote invasion of colorectal cancer cells through PI3K 71. In addition. COX-2 overexpression can affect adhesion properties of intestinal cells 72 and promote matrix metalloproteinase activity and thus cancer invasion 73. Inhibition of COX-2 can antagonize colorectal metastasis in human 74 and in mouse 75 models. c-Met, hepatocyte growth factor receptor, may be transactivated by PGE_2_ via EGFR in case of colorectal cancer 76 which is linked with loss of cell-cell contact and invasion 77. The supporting evidence includes co-existence of β-catenin and c-Met at invasive edge of colorectal tumor 76. COX‐2 may promote carcinogenesis via β1‐integrin 78, 79. In all, these reports indicate that COX-2 indeed actively participate in tumorigenesis. Interestingly, a recent article demonstrates that COX-2 in addition to cancer epithelial cells is also expressed in the cancer stroma cells and in the stroma cells of the adjacent normal tissue and that prostaglandin E2 (the final product of arachidonic acid catalysis by COX-2) is significantly more profoundly expressed in the adjacent normal tissue compared to cancer tissue (p <0.002) 80, suggesting that the role of COX-2 appears to be involved in the early stages of progression of the oncogenetic mechanism of CRC, initially affecting the host (tumor microenvironment) of the tumor in normal cells and later tumor epithelial cells of the tumor itself. The authors suggest that treatment should be given to prevent CRC rather than to suppress this progression especially to colorectal tumors in which the expression was found to be greatest.

## COX-2-promoted carcinogenesis is related to angiogenesis

Suppression of COX-2 inhibits corneal neovascularization in colorectal cancer 81 by promoting production of angiogenic vascular endothelial growth factor (VEGF), a potent angiogenic growth factor 82. In fact, overexpression of COX-2 promotes overexpression of VEGF, which induces tumor angiogenesis in Apc/COX-2 knockout mouse model 83. Deletion of COX-2 gene may result in reduced growth in tumor xenografts and vascular density 84, probably via activation of Rac1 and Cdc42 85. In all, evidence indicates that COX-2 may induce uncontrollable angiogenesis in colorectal cancer.

## Cytokines and other compounds regulating COX-2 signaling

Recent reviews have summarized recent advances in the role of COX-2 and prostaglandin E_2_ in the pathogenesis of colorectal cancer 18, 61, 62. A report suggests that PGE_2_ signaling mediates chronic inflammation in the colorectal microenvironment 86. Cytokines and other compounds such as DCA, IL-1β, tumor necrosis factor α (TNF-α) and lipopolysaccharide (LPS) may promote expression of COX-2 mRNA and protein in human colorectal fibroblasts, profoundly in cancer-associated fibroblasts (CAF) 25-27. When stimulated with the pro-inflammatory cytokines interleukin (IL)-1β or TNF-α, orbital fibroblasts express high levels of COX-2 and synthesized correspondingly high levels of PGE_2_ 87. Powell and colleagues described a population of specialized sub-epithelial “myofibroblasts” with pleiotropic capabilities, including the ability to modulate intestinal secretory responses to inflammatory mediators by releasing PGE_2_ 88. We have noted that IL-1β or TNF-α promotes production of PGE_2_ by as much as 25-fold in human colorectal fibroblasts we obtained from colonoscopies 30. Equivalent or greater increases in COX-2 mRNA and protein expression preceded the increases in PGE_2_ synthesis. We also report that inducible COX-2 expression is substantially more robust in cancer-associated than normal colorectal fibroblasts 28. IL-1β may stimulate expression of COX-2 and production of PGE_2_ synthesis in cancer-associated fibroblasts by activating *COX-2* promoter activities. The rate at which COX-2 mRNA decays can be dramatically retarded *in vitro* by PGE_2_ 89. We believe that this mechanism may be an important contributing factor to the enhanced PGE_2_ synthesis of cancer-associated colorectal fibroblasts. Those reactions are probably through activation of protein kinase C (PKC) 90, suggesting that stromal cells also play an important role in colorectal carcinogenesis. Such activation may be through enhanced production of PGE_2_, and as a result, mediating proliferation, invasion and apoptosis of colorectal cancer cells 25, 27. Such activation of COX-2 is closely associated with increased proliferation and invasiveness in human colorectal epithelial cancer cells 25, 27, 28, because COX-2 inhibitor (NS398) or PKC inhibitor (Bisindoylmalemide I, BIM or Staurosporine, STA) can dramatically down-regulate the proliferation and invasiveness of colorectal epithelial cancer cells 25, 27, 28. Combination of PKC and COX-2 inhibitors can synergistically inhibit melanoma metastasis 91. We have noted that IL-1β and TNF-α induce mRNA overexpression of COX-2 and promote production of PGE2 in human colorectal fibroblasts, especially in CRC-associated strains 27, 92. We have also noted that DCA strongly promotes COX-2 expression and PGE2 production in colorectal cancer fibroblasts *in vitro* 25. Inducible nitric oxide synthetases (iNOS) are also probably involved in the carcinogenesis of colorectal cancer because activation of iNOS by LPS is associated with activation of COX-2 signaling, and inhibition of iNOS by iNOS inhibitor 1400W or iNOS siRNA may nullify production of nitric oxide (NO) and PGE_2_ 26. NO may also promote mRNA production of COX-2 from colorectal cancer cells, for example, HCA7 and HCT116 56. We have reported that deoxycholic acid (DCA) also dramatically promotes COX-2 expression in colorectal cancer fibroblasts *in vitro* 25. The expression and activity of iNOS and COX-2 may also be induced by LPS in those fibroblasts, resulting in increased NO production and COX-2 expression 26. DCA is also a transcriptional activator of COX-2 in esophageal cancer cells 93.

## Nonsteroidal Anti-inflammatory drugs

For decades, a significant progress is achieved to discoveries of effective drugs for CRC. One of those is nonsteroidal anti-inflammatory drugs (NSAID) which inhibit COX-2 94-96. NSAID includes aspirin, ibuprofen, naproxen, nimesulide and sulindac acid. Different NASID may act via different signaling pathways. For example, ibuprofen, indomethacin and naproxen can bind the activity site of COX-2, inhibit its activity reversibly, while aspirin acetylates the activity site of COX-2, attenuating its activity irreversibly. Some NSAID drugs for example, Aspirin, can facilitate the effect of COX-2 inhibitors for treatment of stage III colorectal cancer 97. In fact, Aspirin may reduce colon cancer mortality in women by as much as 50 % 9, 98, 99. Recently, a hybrid drug KSS19, a combination of NSAID refecoxib and cis-stilbene, has been found to be a potent COX-2 inhibitor, which inhibits colon cancer cell growth effectively 100.

Although COX-2 inhibitors are promising candidates for treatment of colorectal cancer, some concerns for treatment of colorectal cancer by COX inhibitors have been raised. For example, an elevated risk of myocardial infarction may be linked to its usage 101. In addition, the extended use of nonselective NSAID is also associated with a number of pathological symptoms, for example, abdominal pain, dyspepsia, gastritis, gastrointestinal bleeding nausea, and perforation of gastroduodenal ulcers 102. Therefore, no major clinical trials of those inhibitors were successfully completed due to concerns of their adverse effect. Anyway, NSAID are effective in certain degrees for prevention and treatment of colorectal cancer. For example, a randomized trial demonstrates that NSAID are preventive for colorectal cancer on the patients with polyps 103, 104. According to the results of large-scale trials, including the Adenomatous Polyp Prevention on Vioxx trial 105, the Adenoma Prevention with Celecoxib trial 104, the Prevention of Colorectal Sporadic Adenomatous Polyps trial 106 and colon polyp prevention trial 107, COXIB are effective for prevention of recurrence from sporadic colon cancer. Regular consumption of NSAID is also helpful for lowing the risk of colorectal, breast, lung and prostate cancer 108. In all, it is still unknown how to prevent the potential risk when COX inhibitors are used for treatment of colorectal cancer.

To decrease the risk from COX inhibitors, many researchers have used low dose of COX inhibitors with other NSAID drugs that target other critical pathways in carcinogenesis. For example, combination of celecoxib with erlotinib (an EGFR tyrosine kinase inhibitor) is more effective to control polyp formation using an ApcMin/+ mice model and to inhibit cancer growth in a xenograft model 109. Celecoxib with erlotinib treatment is more effective for treatment of the advanced non-small cell lung cancer 110. A 5-lipoxygenase inhibitor may inhibit resistant tumor cells to SC-236 (COX inhibitor) and tumor growth in a breast cancer animal model 111. Combined treatment of celecoxib with peroxisome proliferators-activated receptor-γ agonist is better than either alone in a mouse breast cancer model 112. Combination of aromatase inhibitors with celecoxib is better for patients suffering from metastatic breast cancer than either alone 113. Therefore, we should reconsider the prospect of COX inhibitors for treatment of colon cancer.

## Perspective

From previous work on certain types of cancer, COX‐2 may be is a key indicator to predict cancer prognosis. Along with the product PGE_2_, COX-2 is a major stimulator for progression of colorectal cancer. Up to now, the reports from basic and clinical investigations have shown that inhibition of PGE_2_ synthesis by specific COX-2 inhibitors, for example, nonsteroidal anti-inflammatory drugs (NSAIDs) may decrease the risk and improve prognosis of carcinogenesis of various types of cancer including colorectal cancer 64, 114-120. Therefore, we need to strive forward to work out a protocol for successful use of COX-2 inhibitors in clinical applications to colorectal cancers and other types of cancer as well.

## Summary

To summarize, the scientific basis for the current proposal, outlined above, is as follows: fibroblasts from mesenchymal (stromal) layer are the major target of cytokines such as IL1β and TNFα; fibroblasts are the predominant mesenchymal cells; fibroblasts from nonneoplastic colorectal tissue are a potent source of COX-2 expression, which is firmly established as an important factor in colorectal carcinogenesis. We believe that the investigation of COX-2 gene regulation is vital for control of this disease.

## Abbreviation

CAF: cancer-associated fibroblast
COX: Cyclooxygenase
CRC: colorectal cancer
DCA: deoxycholic acid
EGF: epithelial growth factor
EP, FP, IP, TP and DP: prostanoid receptors
IL-1β: Interleukin 1β
iNOS: inducible nitric oxide synthase
LPS: lipopolysaccharide
NF: normal fibroblast
NO: nitric oxide
NSAID: nonsteroidal anti-inflammatory drug
p53: tumor protein p53
PG: prostaglandin
PKA: cAMP-dependent protein kinase
PKC: protein kinase C
TNF: tumor necrosis factor
